# Cell Wall Reinforcements Accompany Chilling and Freezing Stress in the Streptophyte Green Alga *Klebsormidium crenulatum*

**DOI:** 10.3389/fpls.2020.00873

**Published:** 2020-06-24

**Authors:** Philip Steiner, Sabrina Obwegeser, Gerhard Wanner, Othmar Buchner, Ursula Lütz-Meindl, Andreas Holzinger

**Affiliations:** ^1^Department of Biosciences, University of Salzburg, Salzburg, Austria; ^2^Department of Botany, Functional Plant Biology, University of Innsbruck, Innsbruck, Austria; ^3^Ultrastructural Research, Department Biology I, Ludwig-Maximilians-University, Munich, Germany

**Keywords:** cold stress, chilling stress, freezing stress, ultrastructure, cell wall, electron microscopy, tomography, FIB-SEM

## Abstract

Adaptation strategies in freezing resistance were investigated in *Klebsormidium crenulatum*, an early branching streptophyte green alga related to higher plants. *Klebsormidium* grows naturally in unfavorable environments like alpine biological soil crusts, exposed to desiccation, high irradiation and cold stress. Here, chilling and freezing induced alterations of the ultrastructure were investigated. Control samples (kept at 20°C) were compared to chilled (4°C) as well as extracellularly frozen algae (−2 and −4°C). A software-controlled laboratory freezer (AFU, automatic freezing unit) was used for algal exposure to various temperatures and freezing was manually induced. Samples were then high pressure frozen and cryo-substituted for electron microscopy. Control cells had a similar appearance in size and ultrastructure as previously reported. While chilling stressed algae only showed minor ultrastructural alterations, such as small inward facing cell wall plugs and minor alterations of organelles, drastic changes of the cell wall and in organelle distribution were found in extracellularly frozen samples (−2°C and −4°C). In frozen samples, the cytoplasm was not retracted from the cell wall, but extensive three-dimensional cell wall layers were formed, most prominently in the corners of the cells, as determined by FIB-SEM and TEM tomography. Similar alterations/adaptations of the cell wall were not reported or visualized in *Klebsormidium* before, neither in controls, nor during other stress scenarios. This indicates that the cell wall is reinforced by these additional wall layers during freezing stress. Cells allowed to recover from freezing stress (−2°C) for 5 h at 20°C lost these additional cell wall layers, suggesting their dynamic formation. The composition of these cell wall reinforcement areas was investigated by immuno-TEM. In addition, alterations of structure and distribution of mitochondria, dictyosomes and a drastically increased endoplasmic reticulum were observed in frozen cells by TEM and TEM tomography. Measurements of the photosynthetic oxygen production showed an acclimation of *Klebsormidium* to chilling stress, which correlates with our findings on ultrastructural alterations of morphology and distribution of organelles. The cell wall reinforcement areas, together with the observed changes in organelle structure and distribution, are likely to contribute to maintenance of an undisturbed cell physiology and to adaptation to chilling and freezing stress.

## Introduction

The aero-terrestrial green alga *Klebsormidium crenulatum* was chosen for ultrastructural and physiological investigations on freezing stress response due to its adaptation to harsh environmental conditions, such as in the Austrian Alps above 2300 m altitude, where it was isolated (Karsten et al., 2010; Holzinger et al., 2011). By the use of ITS rRNA phylogeny, distinct clades (A–G) were determined within the Klebsormidiales (Rindi et al., 2011). *Klebsormidium crenulatum* belongs to the F-clade, which is characterized by long, strong and thick filaments, the cells are cylindrical, becoming barrel shaped and narrow-square in old filaments (Rindi et al., 2011). The cell walls are initially thin, becoming thick and corrugated in old filaments (Mikhailyuk et al., 2015).

*Klebsormidium* is an abundant member of biological soil crusts and tolerates a broad range of abiotic stresses such as high irradiation, temperature fluctuation and desiccation. As both, desiccation and freezing stress lead to cellular water loss, comparable effects on physiology and structure of *Klebsormidium* are expected. Desiccation stress has been extensively studied in different strains of *Klebsormidium* (e.g., Holzinger et al., 2011; Karsten and Holzinger, 2012; Karsten et al., 2016; Pierangelini et al., 2017), that showed varying capacities to tolerate desiccation. *Klebsormidium crenulatum*, air-dried for 3 h and subsequently rehydrated, showed a recovery of the maximum quantum yield of photosystem II used as a proxy of physiological activity (Karsten et al., 2010). Desiccation stress caused subcellular rearrangements (Holzinger et al., 2011) and cell wall modifications in *K. crenulatum*, with an increase in total callose content (Herburger and Holzinger, 2015). Also cold stress (Rippin et al., 2019; Miguez et al., 2020), freezing stress (Elster et al., 2008; Nagao et al., 2008) and osmotic stress (Kaplan et al., 2012) were previously investigated in different *Klebsormidium* strains. In *K. crenulatum*, plasmolysis occurred after a treatment with 800 mM sorbitol, indicating a very negative osmotic potential (i.e., Ψ = −2.09 MPa; Kaplan et al., 2012). In comparison to that, in the aquatic freshwater alga *Micrasterias denticulata*, plasmolysis occurred after a treatment with 339 mM sorbitol (Affenzeller et al., 2009) suggesting constitutively higher contents of osmotically active substances in *K. crenulatum*, which are likely beneficial for freezing stress tolerance.

Several reports on different higher plants have already indicated that cold stress and in particular freezing stress has severe effects on cell wall structure, cell wall arrangement and modification (Le Gall et al., 2015; Bilska-Kos et al., 2017; Parrotta et al., 2019). Formation of extracellular ice causes dehydration (Pearce, 1999; Stefanowska et al., 1999) which can lead to physical rupture of the cell wall, and subsequently to cell death (Pearce, 1999; Mahajan and Tuteja, 2005). Thus, plants need to develop strategies to endure low temperature scenarios (Janska et al., 2010). Cell wall morphology and rigidity appear to be essential for plants to counteract extracellular freezing (Rajashekar and Lafta, 1996).

Organelle interactions have been frequently observed during stress response in different plant cells (Vartapetian et al., 2003; Liberatore et al., 2016; Tanaka et al., 2017; Mathur et al., 2018; Steiner et al., 2018). Mitochondria and ER interact during autophagy (mitophagy; Minibayeva et al., 2012; Broda et al., 2018) and fusion and fission of mitochondria were reported during hypoxic stress, starvation or UV-irradiation (Logan, 2010; Jaipargas et al., 2015; Mueller and Reski, 2015; Wai and Langer, 2016; Arimura, 2018). ER and ER-plasma membrane contact sites and ER-endosome associations are also involved in cellular stress response of eukaryotic cells and have been described in numerous species and different cell types (Hepler et al., 1990; Staehelin, 1997; Stefano et al., 2015; McFarlane et al., 2017; Wang et al., 2017). ER and ER-organelle interactions seem to play an important role in general stress response, yet structural and physiological alterations during low temperature stress in plants are widely unknown.

In the present study, adaptation strategies to cold stress subdivided in chilling- (4°C) and extracellular freezing (−2°C and −4°C) stress were investigated in *K. crenulatum*, with different 2-D and 3-D electron microscopic methods in correlation with physiological experiments. Moreover, to test for the dynamics of the resulting changes, recovery experiments were performed, where frozen cells were allowed to recover and kept at 20°C for 5 h. We hypothesized that chilling and freezing treatments lead to dynamic changes in the cellular ultrastructure and cell wall architecture that might support the physiological adaptations to cold temperatures.

## Materials and Methods

All applied chemicals in the present study were purchased from Roth (Karlsruhe, Germany) and Sigma-Aldrich (Vienna, Austria) except stated differently.

### Cultivation of *Klebsormidium crenulatum*

The freshwater green alga *Klebsormidium crenulatum* was cultivated in Erlenmeyer flasks, containing 100 ml of 3 N MBBM medium [Starr and Zeikus, 1993, Bold’s basal medium (BBM) modified by addition of triple nitrate concentration] during a light cycle of 14 h at 20°C and a dark cycle of 10 h at 20°C. The light intensity was chosen between 100 and 150 μmol photons⋅m^–2^⋅s^–1^. Several filaments of *Klebsormidium* were subcultured every 6 weeks. Approximately 4–5 weeks old filaments were used for the experiments.

### Freezing Samples in the Automatic Freezing Unit (AFU)

Low temperature preparation of *Klebsormidium* was performed in an AFU (for more details see Buchner et al., 2020) prior to high pressure freezing (HPF, see section “Preparation for TEM and FIB-SEM”). In the AFU, *Klebsormidium* was adapted to 4°C from 20°C with −8°C⋅h^–1^ and subsequently cooled down with −2°C⋅h^–1^ (starting at 4°C). Freezing was induced via transfer of ice crystals to the sample in the specimen holder (for details see Buchner et al., 2020). For each final temperature (−2 and −4°C) three independent biological replicates (*n* = 3) were used. As a recovery experiment, *Klebsormidium* cells frozen at −2°C, as described above, were allowed to thaw with a rate of 2°C⋅h^–1^ and kept at 20°C for 5 h prior to HPF.

### Polarographic Oxygen Measurements

Photosynthetic oxygen measurements of *Klebsormidium* controls at 20°C and filaments after 4°C chilling stress were performed by polarographic oxygen determination (Hansatech, King’s Lynn, United Kingdom) during six light (200 μmol photons⋅m^–2^s^–1^) and dark cycles to investigate their respiratory and photosynthetic efficiency during 4°C chilling stress compared to 20°C standard conditions. O_2_ production was measured in algae after 1, 24 h and 3 weeks of chilling stress. Each approach was performed with three independent biological replicates (*n* = 3).

### Preparation for TEM and FIB-SEM

*Klebsormidium* filaments were directly transferred into the specimen holder for high pressure freeze fixation (HPF). Samples from control treatment (20°C), chilled (4°C) and the frozen samples (see section “Freezing Samples in the Automatic Freezing Unit”) were used in three independent biological replicates (*n* = 3) each. The HPF procedure was performed with a Leica EM PACT HPF device (Leica Microsystems, Vienna, Austria). The cooling rate for the HPF was steady above 12000°C⋅s^–1^ at approximately 2040 bar. After this process, *Klebsormidium* was transferred into a Leica EM AFS (Leica Microsystems, Vienna, Austria) for cryo-substitution. The substitution medium contained 2% OsO_4_ (1% OsO_4_ for immuno-TEM) and 0.05% uranyl acetate in anhydrous acetone. Samples were embedded in Agar low viscosity resin (LV resin, VH1 and VH2 hardener and LV accelerator, Agar Scientific, Essex, United Kingdom) for standard TEM and in LR-White (London Resin Company Ltd., Theale, United Kingdom) for immuno-TEM. Thereafter, sectioning for TEM imaging was carried out with a Leica UC7 Ultramicrotome or a Reichert Ultracut S (Leica AG, Vienna, Austria) and ultrathin sections, either unstained or counterstained with 1% uranyl acetate and Reynold’s lead citrate (2.66%), were collected on copper grids with a thin layer of Formvar.

All preparation steps given above were carried out in the same way for FIB-SEM tomography, except the last embedding step (for details see Wanner et al., 2013). There, single strings of *Klebsormidium* were gently spread on top of micro-scale microscope slices (neoLab Migge GmbH, Heidelberg, Germany) to ensure that a thin coat of epoxy resin covered the single strings. Smaller parts of the microscope slices were mounted on stubs and coated with carbon for lateral milling with the FIB (Ga-ion beam).

### 2-D TEM and 3-D TEM Tomography

2-D TEM was performed either in a LEO 912 AB Omega TEM or in a Libra 120 TEM (both Zeiss, Oberkochen, Germany) operated at 80 kV. 3-D TEM tomography was done in a LEO 912 AB Omega TEM at 120 kV. Images were always filtered at zero energy loss and were captured with a TRS (Tröndle Restlicht Verstärker Systeme, Moorenweis, Germany) 2k Slow-Scan CCD camera at both TEMs employed.

For TEM tomography, Formvar coated grids with approximately 200 nm sections were incubated in 2 μl of 15 nm colloidal gold particles (BBI Solutions, Cardiff, United Kingdom) on both grid sides for subsequent alignments. During the tomographic process, samples were automatically tilted between −70° and +70° with an increment of 1°. Acquisition and calibration was performed by iTEM Software (Olympus Soft Imaging Solutions GmbH, Münster, Germany). The tilting series of the TEM tomography were first stack aligned with iTEM several times. For the actual alignment with IMOD (University of Colorado, Denver, CO, United States), a conversion with ImageJ (Schneider et al., 2012) was done. Segmentation was carried out manually with Amira Software (Visualized Sciences Group, Hilsboro, OR, United States).

#### Measurements of ER Cisternae

To obtain an overview of the enlargement of ER cisternae during freezing stress, single ER cisternae were measured in *Klebsormidium*, extracellularly frozen at −4°C in comparison to *Klebsormidium* controls at 20°C. In total, 50 control cells at 20°C (from three independent biological replicates, *n* = 3) were compared to 50 cells after −4°C ice induction (from three independent biological replicates, *n* = 3). For the measurement, the largest diameter of a smooth ER cisternae was measured in each cell.

### FIB-SEM Tomography

In order to enable visualization of larger volumes of *Klebsormidium* filaments and to obtain 3-D insight into the arrangement of the cell wall and additional cell wall layers, FIB-SEM was employed in addition to 2-D and 3-D TEM. For FIB-SEM application, the “slice and view” procedure was implemented with a Zeiss Auriga 40 crossbeam workstation (Carl Zeiss Microscopy, Oberkochen, Germany). Focused ion beam (FIB) milling (0.5–2 nA) of the Ga-emitter was responsible for the “slice” process. The “view” procedure was carried out and recorded by the scanning electron microscope (SEM) with an aperture of 60 μm in high-current mode at 0.5 kV of the in-lens EsB detector. The semi-automatic alignment and the manual segmentation of the FIB-SEM images were carried out with Amira Software.

### Immuno-TEM

Immunogold labeling with TEM analysis was performed to determine the cell wall components, including the additional cell wall reinforcement areas attached on the inner surface of the cell wall of *Klebsormidium* during freezing stress at −4°C. For this approach, the following monoclonal antibodies were applied: JIM 5 against unesterified pectins, JIM 7 against methyl esterified pectins, JIM 13 and JIM 15 against arabinogalactan proteins, LM 7 against homogalacturonan, LM 8 against xylogalacturonan, LM 10 and LM 11 against unsubstituted and low substituted xylan, CCRC-M1 against xyloglucan and rhamnogalacturonan I, BG 1 and callose antibody (400-2; Biosupplies Australia Pty Ltd.) against β-_D_-1,3-glucans, Lectin [soybean agglutinin, Sigma-Aldrich (Vienna, Austria)] against N-acetyl-_D_-galactosamine, 2F4 against homogalacturonan, Mac 207 against arabinogalactan and Xgn against xyloglucan. All primary antibodies were purchased from Plant Probes (Leeds, United Kingdom), unless stated differently. For each approach, ultrathin sections were blocked in 50 mM Trizma-buffer (pH 6.8) with 2% BSA (bovine serum albumin) and 0.1% Tween 20 (polyoxyethylene sorbitan monolaurate) for 40 min. Sections were incubated overnight at 4°C in the primary antibody. Each antibody was first tested in a low dilution ratio of 1:5 with Trizma-buffer (50 mM, pH 6.8, with 2% BSA and 0.1% Tween 20) to guarantee recognition of even low concentrated epitopes. The primary antibodies were further diluted in Trizma-buffer when necessary (for specific dilutions see Table 1). Thereafter the sections were washed in Trizma-buffer four times and then incubated in the secondary antibody in Trizma-buffer [for second antibodies see Table 1, purchased from Sigma-Aldrich (Vienna, Austria)], conjugated with 10 nm gold particle at room temperature for 1 h. As a last step, grids were washed with the buffer (see above), followed by distilled water and were then dried. As negative controls, sections were treated as described above, but the first antibody was omitted. This procedure was performed with all antibodies except Lectin. For Lectin, ultrathin sections were first blocked with 10 mM PBS (0.5% BSA, 0.05% Tween 20) for 40 min and were then transferred to the Lectin marker. As a last step, the Lectin marker was incubated overnight at 4°C. All labeling methods were performed with extracellularly frozen *Klebsormidium* samples at −4°C prior to TEM preparation (see section “Preparation for TEM and FIB-SEM”) and were subsequently analyzed in the TEM (see section “2-D TEM and 3-D TEM Tomography”).

**TABLE 1:** Antibodies that were used for analysis of the cell wall and cell wall reinforcement areas (CWRAs) in *Klebsormidium* (extracellularly frozen at −4°C), subdivided in first antibody, second antibody, specification/antigen, labeling and type of antibody.

**First Antibody**	**Second Antibody**	**Specificity/Antigen**	**Labeling**	**Type**
JIM 5 *1:1*	Anti-rat IgG *1:40*	Homogalacturonan, unesterified pectins	No labeling	Monoclonal
JIM 7 *1:1*	Anti-rat IgG *1:40*	Homogalacturonan, methyl-esterified pectins	No labeling	Monoclonal
JIM 13 *1:10*	Anti-rat IgG *1:40*	Arabinogalactan proteins	No labeling	Monoclonal
JIM 15 *1:5*	Anti-rat IgG *1:40*	Arabinogalactan proteins	No labeling	Monoclonal
LM 7 *1:5*	Anti-rat IgG *1:40*	Homogalacturonan	No labeling	Monoclonal
LM 8 *1:5*	Anti-rat IgG *1:40*	Xylogalacturonan	No labeling	Monoclonal
LM 10 *1:10*	Anti-rat IgG *1:40*	Unsubstituted and low substituted xylan	No labeling	Monoclonal
LM 11 *1:5*	Anti-rat IgG *1:40*	Unsubstituted and low substituted xylan and arabinoxylan	Overall cell wall	Monoclonal
CCRC-M 1 *1:5*	Anti-rat IgG *1:40*	Xyloglucan, rhamnogalacturonan I	No labeling	Monoclonal
BG 1 *1:40*	Anti-mouse IgG *1:40*	(1,3),(1,4)-β-glucans	No labeling	Monoclonal
Callose *1:50*	Anti-mouse IgG *1:60*	(1,3)-β-glucans	Rare, in thick cross wall areas	Monoclonal
Lectin *1:10*	None	N-acetyl-D-galactosamine	No labeling	Monoclonal
2F4 *1:10*	Anti-mouse IgG *1:40*	Homogalacturonan	No labeling	Monoclonal
Mac 207 *1:5*	Anti-rat IgG *1:40*	Arabinogalactan proteins	No labeling	Monoclonal
Xgn *1:5*	Anti-rat IgG *1:40*	Xyloglucan	No labeling	Monoclonal

### Confocal Laser Scanning Microscopy and Aniline Blue Staining

Confocal Laser Scanning Microscopy (CLSM) was performed with a Leica TCS SP5 AOBS (Leica Microsystems, Wetzlar, Germany), coupled with a Leica DMI 6000 CX inverted microscope. Based on the results of a previous study (Herburger and Holzinger, 2015), callose was stained with 2% aniline blue for 2 h (adapted from Herburger and Holzinger, 2016) and was subsequently investigated at the CLSM. Visualization was obtained with an excitation of 405 nm (405 diode, UV-laser) and an emission bandwidth between 425 and 548 nm. The aniline blue staining and subsequent CLSM analysis was performed with *Klebsormidium* controls at 20°C and samples that were cooled down from 20°C to 4°C with −8°C⋅h^–1^ and from 4°C to −2°C with −2°C⋅h^–1^. Freezing was induced at −2°C and *Klebsormidium* was kept in extracellularly frozen state for 1 h. The alga was thawed at 4°C, stained with aniline blue at 4°C and was then investigated by the CLSM at room temperature. Analysis of the fluorescence intensity was performed with the quantification function in the Leica application suite X software (Leica Microsystems, Wetzlar, Germany). From three independent biological replicates (*n* = 3), filaments were selected and the fluorescence intensity of the first six cross walls within a filament were measured.

### Statistical Data Analysis

Descriptive statistical data analysis and comparisons of mean values by *t*-Test (in the case of ER lumen measurements and fluorescence intensity measurements of the aniline blue staining) or by analysis of variance (one-way ANOVA) followed by Duncan’s multiple range test (in the case of polarographic oxygen measurements), were performed with statistics software (IBM SPSS V.26.0, SPSS Inc., Armonk, NY, United States). For all statistical tests normal distribution and variance homogeneity was checked by the Shapiro–Wilk and the Levene test, respectively, a significance level of *p* < 0.05 was used.

## Results

### Photosynthesis and Dark Respiration During Chilling Stress at 4°C in Comparison to 20°C Controls

**FIGURE 1:**
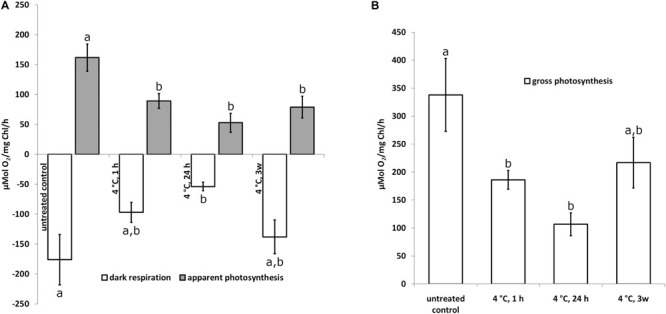
Apparent photosynthesis **(A)** gray bars, dark respiration **(A)** open bars and gross photosynthesis **(B)** open bars of *Klebsormidium* after 1, 24 h and 3 weeks of chilling stress at 4°C. Bars represent means of three independent biological replicates (*n* = 3). Standard error of the mean is displayed with a line. Different letters (a, b) indicate significant differences between mean values (*p* < 0.05; Duncan’s multiple range test).

To investigate the physiological status of *Klebsormidium* during chilling stress, polarographic oxygen measurements were performed during 4°C chilling stress. Our results show a statistically significant (*p* < 0.05) decrease of photosynthesis (apparent and gross photosynthesis) after 1 h of 4°C chilling stress when compared to control cells (Figures 1A,B). Dark respiration was significantly (*p* < 0.05) different from the control after 24 h of 4°C chilling stress (Figure 1A). The respiration and photosynthesis values, however, were not significantly different when 1 h, 24 h or 3 weeks of 4°C chilling stress were compared (Figures 1A,B). As there are no significant differences between the control and the 3 weeks chilling (4°C) stressed cells (Figure 1B), an acclimation process is possible.

### Structural Alterations During Chilling and Freezing Stress in Comparison to 20°C Controls (2-D TEM)

**FIGURE 2:**
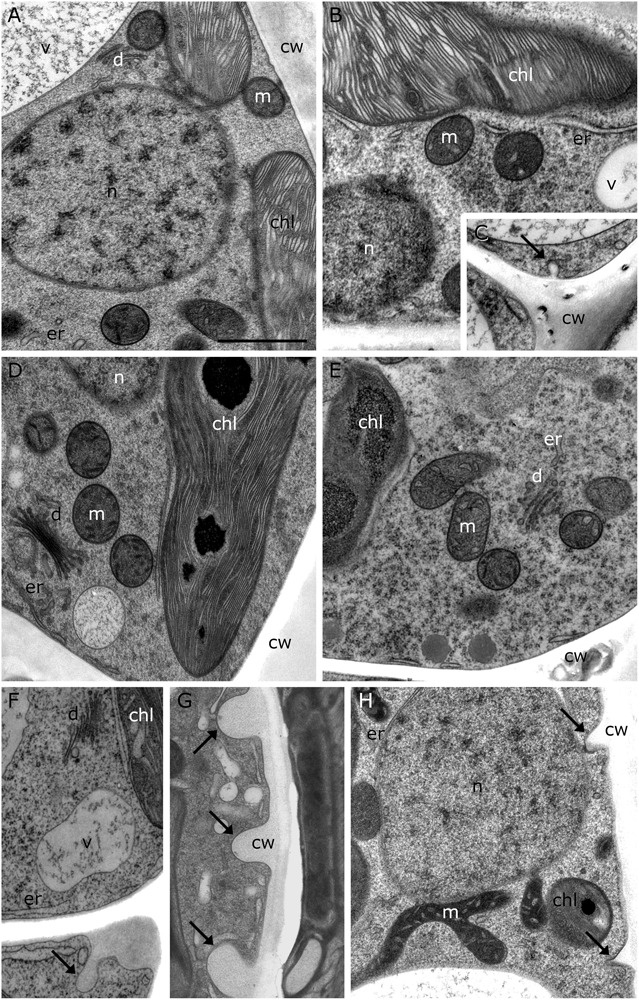
TEM micrographs of *Klebsormidium* during cold stress in comparison to control at 20°C. **(A)** Control cell at 20°C shows solitary distributed spherical mitochondria, regular ER cisternae and undisturbed thylakoid structure. **(B)** Cell after 1 h, 4°C chilling stress with increased ER cisternae and **(C)** showing a small cell wall plug (CWP). **(D)** Cell after 24 h, 4°C chilling stress with increased ER cisternae. **(E)** Cell after 3 weeks, 4°C chilling stress shows aggregation of mitochondria and enlarged ER. **(F)** After –2°C freezing stress, ER cisternae are increased and cell wall plugs (CWPs; arrow) are present. **(G)** After –4°C freezing stress, extended CWPs (arrows) are present. **(H)** –4°C freezing treatment induces elongation and fusion of mitochondria and the number and size of CWPs (arrows) increase. m, mitochondria; cw, cell wall; v, vacuole; chl, chloroplast; ER, endoplasmic reticulum; d, dictyosomes; scale bar (1 μm) applies to all images.

**FIGURE 3:**
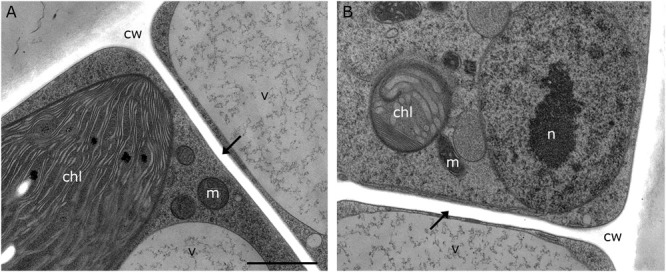
TEM micrographs of *Klebsormidium* kept at 20°C for 5 h after recovery from freezing at –2°C. **(A,B)** Recovery cells show cell walls and cross walls (arrows) without appearance of cell wall plugs or additional cell wall layers. cw, cell wall; chl, chloroplast; m, mitochondrion; v, vacuole; n, nucleus; scale bar (1 μm) applies to both images.

20°C controls of *Klebsormidium* show a homogenous cell wall and random organelle distribution such as solitary distributed and spherical mitochondria and undisrupted thylakoid structure and arrangement of the chloroplast (Figure 2A and Table 2). Moreover, only unbloated ER cisternae and unimpaired dictyosomes are found in 20°C controls (Figure 2A and Table 2). During chilling stress (4°C), the cell wall- and most of the organelle structures appear similar to the 20°C controls. However, mitochondria start to aggregate and ER cisternae and the thylakoid structure is slightly enlarged (Figures 2B–E and Table 2). Moreover, inward orientated cell wall plugs (CWPs, approximately 200 nm in length) appear infrequently during chilling stress at 4°C (Figure 2C; arrow and Table 2) at the corner areas of the cell wall, which are not present in controls. When these CWPs appear, the cytoplasm is not retracted from the cell wall. When freezing stress is induced (−2°C), size and number of CWPs increase mostly at corner areas of the cross wall (Figure 2F; arrow and Table 2). When freezing stress is induced at −4°C, CWPs are frequently found along the inner side of the entire cell wall but most prominently at corner areas of the cross walls (Figures 2G,H; arrows and Table 2), yet the cytoplasm is still not retracted from the cell wall. During freezing stress (−2°C and −4°C) mitochondria elongate, fuse and aggregate to local clusters. Dictyosomes reduce their number of cisternae, smooth ER cisternae significantly (*p* < 0.05) increase their volume when compared to controls (Supplementary Figure S1 and Table 2) and the thylakoids of the chloroplast are dilated (Figures 2F–H and Table 2). *Klebsormidium* cells, allowed to recover from freezing stress (−2°C) and thawed to 20°C, did not show CWPs (Figures 3A,B).

**TABLE 2:** Summary of ultrastructural alterations in *Klebsormidium crenulatum* after 1, 24 h and 3 weeks chilling stress (4°C), freezing stress (−2 and −4°C) in comparison to controls at 20°C.

**Object**	**Cell wall**	**Mitochondria**	**Dictyosomes**	**ER**	**Chloroplast**
*Klebsormidium crenulatum*, 20°C control	Cell wall without structural alterations	Spherical shaped, solitary distributed	No alterations of cisternae	Regular, unbloated ER	Regular thylakoid structure
*Klebsormidium crenulatum*, 4°C, 1 h chilling stress	Rare appearance of minor CWPs (inwards)	Spherical shaped, solitary distributed	No alterations of cisternae	Enlarged ER	Minor dilation of thylakoids
*Klebsormidium crenulatum*, 4°C, 24 h chilling stress	Rare appearance of minor CWPs (inwards)	Occasional aggregation	No alterations of cisternae	Enlarged ER	Minor dilation of thylakoids
*Klebsormidium crenulatum*, 4°C, 3 weeks chilling stress	Rare appearance of minor CWPs (inwards)	Aggregation	No alterations of cisternae	Enlarged ER	Minor dilation of thylakoids
*Klebsormidium crenulatum*, −2°C freezing stress	CWPs (2-D) and CWRAs (3-D) at cross walls, corner areas and lateral cell wall (inwards)	Occasional aggregation and fusion	Degradation of cisternae	Bloated and enlarged ER	Dilation of thylakoids
*Klebsormidium crenulatum*, −4°C freezing stress	Increased CWPs (2-D) and CWRAs (3-D) at cross walls, corner areas and lateral cell wall (inwards)	Elongation, occasional aggregation and fusion	Degradation of cisternae	Enlarged ER by half in comparison to control (statistics in Supplementary Figure S1), network with other organelles, close vacinity to cell wall	Dilation of thylakoids

### Cell Wall Alterations and Organelle Aggregations During −4°C Freezing Stress (3-D TEM-Tomography)

**FIGURE 4:**
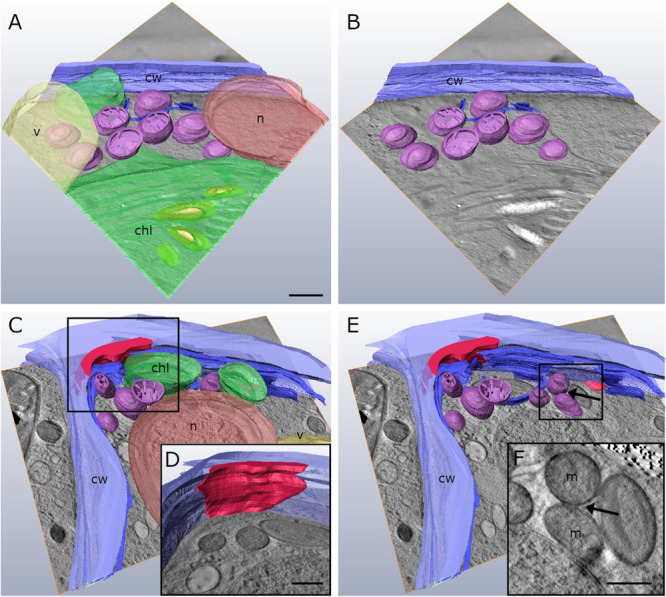
Reconstruction from TEM tomography series of *Klebsormidium* control at 20°C **(A,B)** in comparison to extracellularly frozen *Klebsormidium* cell at –4°C **(C–E)**. **(D)** Alternate angle of TEM tomogram (scale bar: 500 nm) visualizing enormous CWRA (magenta) indicated in panel **(C)**. **(F)** TEM section (scale bar: 500 nm) of TEM tomography shows fusion of mitochondria (arrow) and extended ER cisternae in extracellularly frozen *Klebsormidium* cell at –4°C, indicated in panel **(E)**. purple, mitochondria; blue, ER; yellow, starch grains; magenta, additional cell wall layer representing reinforcement area; cw, cell wall; v, vacuole; chl, chloroplast; n, nucleus; m, mitochondria; TEM tomograms **(A–C,E)** were recorded with equal magnification (scale bar: 500 nm).

Our 2-D TEM findings in *Klebsormidium* cells after freezing stress indicate alterations of the cell wall structure and an increase in organelle aggregation as well as changes in their morphology. For appropriate structural interpretation, these alterations were investigated by high-resolution TEM tomography in order to obtain a 3-D insight into freezing induced structural changes. 3-D TEM tomograms of an untreated *Klebsormidium* control at 20°C display a homogenous cell wall structure, solitary distributed mitochondria and minor appearance of ER cisternae (Figures 4A,B and Table 2). In contrast, reconstruction of an extracellularly frozen (−4°C) *Klebsormidium* cell shows extended CWPs, aggregating to inward facing cell wall layers (Figures 4C–E and Table 2). Our 3-D TEM visualization depicts an extended area of additional cell wall layers located at the corner of the cell indicated in magenta (Figure 4D). Neither CWPs nor additional cell wall layers are present in controls of *Klebsormidium* at 20°C (Figures 4A,B and Table 2). In extracellularly frozen *Klebsormidium* cells at −4°C, mitochondria differ slightly in size and shape in comparison to controls at 20°C. During freezing stress, fusion of single mitochondria is observed (Figures 4E,F, arrows and Table 2) and extended smooth ER cisternae are forming networks between mitochondria, chloroplasts and the nucleus in close position to the cell wall (Figure 4C and Table 2). In a previous study (Nagao et al., 2008), an overall increase of the chloroplast was reported in *Klebsormidium flaccidum* after 2 days chilling stress at 2°C by analyzing 2-D TEM data. In our study, an increase in chloroplast size of *Klebsormidium crenulatum* could neither be observed during chilling nor during freezing stress after 2-D and 3-D TEM investigation.

### Cell Wall Alterations During −2°C Freezing Stress in FIB-SEM Tomography

**FIGURE 5:**
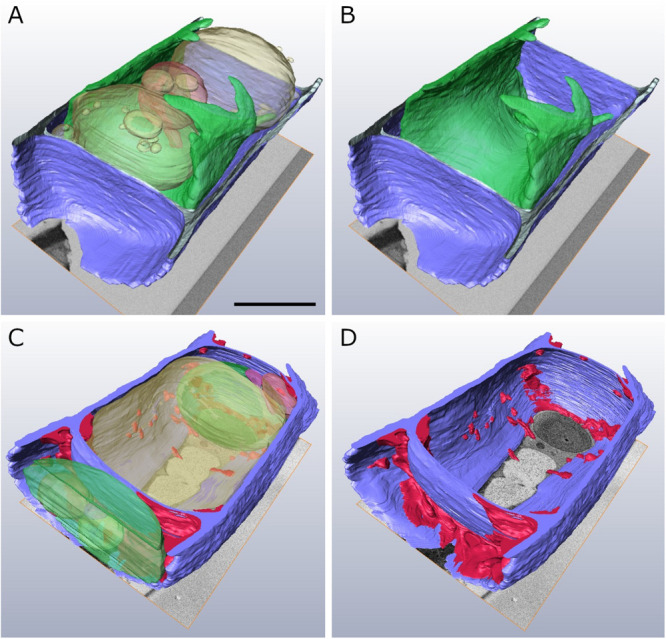
Reconstructed FIB-SEM sections of *Klebsormidium* control at 20°C **(A,B)** in comparison to extracellularly frozen *Klebsormidium* cell at –2°C **(C,D)**. CWRAs of the cell corners (magenta) are clearly visible in the frozen cell at –2°C. blue, cell wall; magenta, additional cell wall layers representing reinforcement areas; transparent yellow, vacuole; transparent green, chloroplast; transparent red, peroxisome; scale bar (4 μm) applies to all images.

In order to obtain a comprehensive view of the 3-D distribution of the additional cell wall layers in our TEM tomograms within a whole *Klebsormidium* cell, 3-D FIB-SEM tomography was performed. This provides important information in addition to TEM tomography as this technique allows depiction of large volumes or whole cells. By this method, the presence of inward facing additional cell wall layers during −2°C freezing was confirmed. The reconstruction of a *Klebsormidium* control cell at 20°C shows an overall homogenous cell wall structure without any structural alterations or occurrence of CWPs or cell wall layers. It also depicts the parietal chloroplast, with the shape of an open ring that covers the other organelles in controls at 20°C (Figures 5A,B and Table 2). After −2°C freezing stress, the reconstruction shows an enormous amount of additional cell wall material. As most of it is located at the cell corners, it is likely that it reinforces the cell wall during freezing stress, corroborating our TEM tomography investigations. Some of these additional cell wall layers are also found at the lateral side of the cell wall (Figures 5C,D and Table 2).

### Callose and Xylan Are Constituents of the Cell Wall, but Do Not Label CWPs and Additional Cell Wall Layers (Immuno-TEM)

**FIGURE 6:**
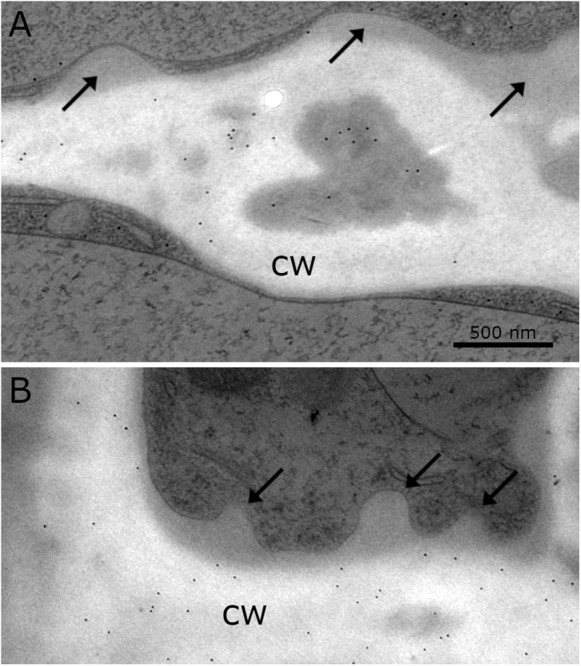
Distribution of gold particles after immuno-staining with the antibodies for **(A)** callose and **(B)** xylan (LM 11) in the cell wall (cw) of *Klebsormidium*, frozen at –4°C, no labeling of CWRAs (arrows). **(A)** Thick cross wall of *Klebsormidium* with electron dense area (asterisk) prior to fragmentation and attached additional cell wall layers (arrows). **(B)** Longitudinal cell wall and cross wall with cell wall plugs (CWPs) (arrows). Gold particles measure 10 nm in diameter; scale bar (500 nm) applies to both images.

Immuno-TEM gold staining shows specific labeling of callose and xylan in the cell wall of *Klebsormidium* by callose and LM 11 antibodies, respectively. The callose antibody labels thickened cross walls (Figure 6A and Table 1). All other tested antibodies (Table 1) did not result in cell wall staining in *Klebsormidium*. When the first antibodies were omitted as negative controls, no staining occurred, as also previously shown for the callose antibody (Herburger and Holzinger, 2015). Some cross walls comprise electron dense areas (Figure 6A, asterisk) in the center or in the corner area of the cross wall which are also labeled by the callose antibody. These areas were reported before as spaces that occur in *Klebsormidium crenulatum* prior to fragmentation of two neighboring cells (Mikhailyuk et al., 2015). Thin cross walls and lateral cell walls are not labeled by the callose antibody. Furthermore, the additional cell wall layers did not show labeling by the callose antibody (Figure 6A; arrows). The xylan antibody recognizes epitopes in the entire cell wall of *Klebsormidium*, both in cross walls and longitudinal walls (Figure 6B and Table 1). However, it does also not label CWPs and additional cell wall layers (Figure 6B; arrows).

### Visualization of Callose by Aniline Blue Staining

**FIGURE 7:**
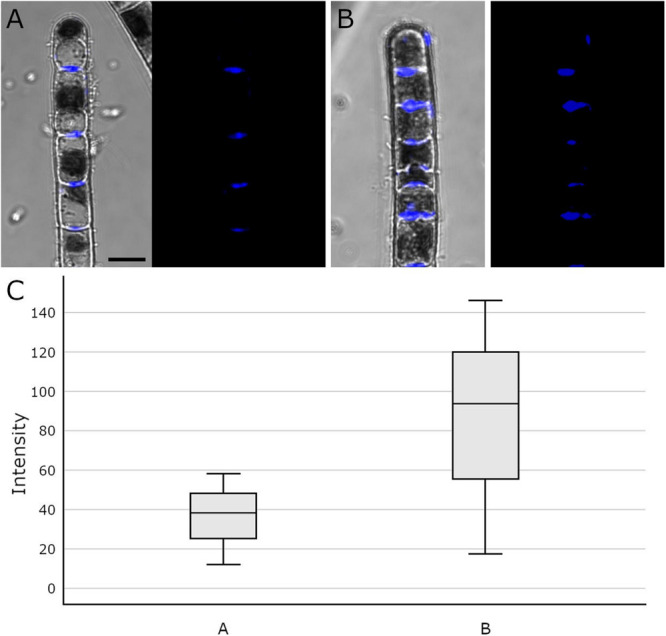
Confocal Laser Scanning micrographs (merged micrograph and pure staining in blue) of aniline blue stained *Klebsormidium* control at 20°C **(A)**, after freezing at –2°C **(B)** and **(C)** boxplots of the fluorescence intensity distribution of the aniline blue staining. **(A)** CLSM images of *Klebsormidium* control at 20°C, with abundant callose (blue) in the center of cross walls. **(B)** CLSM images of *Klebsormidium* after –2°C freezing stress with abundant callose in thickened cross walls and minor callose in the tip area. **(C)** boxplots showing the median and interquartile range (IQR) from three independent biological replicates (*n* = 3), whiskers represent 1.5 IQR, *t*-Test did not show significant differences. Scale bar (5 μm) applies to A,B.

Aniline blue staining was investigated by CLSM to detect the distribution of callose in cross cell walls of *Klebsormidium* filaments. This method allows staining of the total amount of callose in the cell walls and not only of specific free epitopes as in immuno-TEM. We found abundant callose (in blue) at thickened cross walls of untreated controls at 20°C (Figure 7A) and filaments of *Klebsormidium* after freezing stress at −2°C and subsequent thawing for CLSM implementation (see section “Confocal Laser Scanning Microscopy and Aniline Blue Staining”; Figure 7B). Cells frozen at −2°C show individual cross walls with drastically increased callose staining (Figure 7B). Boxplots (Figure 7C) show a higher median and variance of the fluorescence signal in −2°C frozen *Klebsormidium* (mean value: 85.7 + 37.4 SE) when compared to the 20°C controls (mean value 36.2 + 13.3 SE). Nevertheless, no significant differences could be detected by *t*-Test.

## Discussion

The streptophyte green alga *Klebsormidium crenulatum* is highly adapted to unfavorable environmental conditions at its extreme habitats. As a survival strategy, the filamentous alga has developed adaptation mechanisms to withstand desiccation, high irradiation and rapid temperature fluctuation. During desiccation for example, the flexible cross walls of *Klebsormidium* have to withstand physical pressure due to water loss (Holzinger et al., 2011).

In the present study, 2-D TEM investigations showed infrequent CWPs that appeared in single filaments of *Klebsormidium*, located at the corner area of the lateral cell wall and the cross wall, even during chilling stress at 4°C. To our knowledge, similar alterations of the cell wall during cold stress have not been reported in plant or algal cells so far. The infrequent CWPs observed during chilling stress (at 4°C) increased in size and were found in larger parts of the cross wall and corner area of the cell wall when freezing stress was induced at −2°C. When freezing was induced at −4°C, most *Klebsormidium* cells were coated with additional layers of cell wall material, that were interpreted as cell wall reinforcement areas (CWRAs). Our 3-D TEM tomograms and FIB-SEM reconstructions suggest that the additional cell wall layers function as cell wall reinforcements during freezing stress, as they were most prominently attached to the corner areas of the cell wall. We suggest, that the CWRAs counteract the physical pressure of the frozen surrounding medium, to avoid freeze-induced cell wall rupture, as reported before (Rajashekar and Lafta, 1996).

Due to the increase of CWPs to CWRAs from chilling to freezing stress, it was our first hypothesis that these cell wall reinforcements could support the rigidity of the xylan rich cell walls. This view is also supported by recovery experiments, where thawing (to 20°C with a rate of 2°C⋅h^–1^ and then keeping the samples for 5 h at this temperature) frozen samples (−2°C) leads to a total loss of CWPs or CWRAs, suggesting a stress induced dynamic occurrence of these structures. The CWPs and CWRAs might help the cell to cope with physical pressure and dehydration during ice formation. A previous study with *Klebsormidium* showed that the alga increased the amount of callose in the cross wall areas during desiccation stress (Herburger and Holzinger, 2015). It was assumed that the alga therefore enables more flexibility and the filaments decreased their volume through shrinkage to counteract dehydration. In our study, we have observed a similar effect, when callose was more abundant in individual thickened cross walls of frozen cells, however, this effect was not statistically significant. Additionally, in some of these thickened cross walls, electron dense areas were observed in the center and in the corner areas of the cell wall by 2-D TEM depiction. Similar structural alterations of the cell wall were reported before in *Klebsormidium crenulatum* (Mikhailyuk et al., 2015). In the latter study, spaces in the cell wall occurred prior to fragmentation of two neighboring cells of *Klebsormidium crenulatum* kept at 20°C. We assume that an increase of callose supports the required flexibility of the cross walls to counteract dehydration during freezing stress. This was accompanied by an increase of CWRAs, presumably to support the overall rigidity of the cell wall to withstand the physical pressure of extracellular ice. Besides the determination of callose in the cross wall areas and xylan in the entire cell wall, the components of the CWRAs are yet unknown. As all antibodies used for immuno-gold TEM analysis in the present investigation did not label the CWRAs, we do not have direct evidence that they contain cell wall material.

However, in a study by Takahashi et al. (2019) acclimation of *Arabidopsis* to cold and sub-zero temperatures was accompanied by extensive changes in cell wall amount, composition and structure. These changes were related by a proteomic approach to the accumulation of cell wall modifying enzymes such as pectin methylesterases, pectin methylesterase inhibitors and xyloglucan endotransglucosylases/hydrolases in the extracellular matrix. In *Klebsormidium*, we have experimental evidence for xyloglucan:xyloglucan endotransglucosylase (XET) activity (Herburger et al., 2018), as well as novel transglycosylation activities between xyloglucan and xylan, and xyloglucan and galactomannan were identified *in vitro* (Herburger et al., 2018). Thus, it might be possible that the chilling- and freezing induced additional layers found in *Klebsormidium* are modified by these enzymes in a way that they are not recognized by the standard set of antibodies applied in this study. One possible way to test this would be the application of e.g., XET to *Klebsormidium* and then investigate by immuno-gold TEM if the binding activities of the positively tested antibodies are changed.

In addition to the alterations of the cell wall, further ultrastructural adaptation mechanisms of *Klebsormidium* were observed during chilling and freezing stress. During chilling stress at 4°C, minor ultrastructural alterations were observed. Mitochondria started to aggregate and elongate, thylakoid membranes appeared slightly dilated and an enlarged ER network was observed in close vicinity to the chloroplast. However, dictyosomes showed no visible alterations in size, shape or vesicle production during chilling stress.

In freezing stressed *Klebsormidium* filaments (−2°C and −4°C), mitochondria aggregated and fused to small clusters. Similar aggregation was observed in the unicellular freshwater alga *Micrasterias denticulata* during ionic stress (Steiner et al., 2018). There, mitochondria aggregated and fused to large clusters when cells were stressed with potassium chloride (Steiner et al., 2018). Alterations of morphology and distribution of mitochondria were reported before as hallmarks for stress in eukaryotic cells (Vartapetian et al., 2003; Scott and Logan, 2008; Welchen et al., 2014) and correlate with our investigations of the ultrastructure in *Klebsormidium* during cold stress. Furthermore, enlarged smooth ER cisternae aggregated to multi-organelle-networks with mitochondria, nucleus and chloroplast during −4°C freezing stress in *Klebsormidium* (Figure 4C). In this respect the results in *Klebsormidium* correspond well to those obtained in *Micrasterias* during ionic stress, where stress-induced mitochondrial networks were also in contact with degrading dictyosomes or extensive ER cisternae (Steiner et al., 2018). In the present study, the enlarged smooth ER cisternae were located in close position to the corner area of the cell wall and the CWRAs during freezing stress in *Klebsormidium*. We assume that the close position of the enlarged smooth ER to the cell wall and the CWRAs favors the stabilization process by calcium (Ca^2+^) regulation. The increased calcium concentration therefore supports the fusion of vesicles and subsequently the fusion of vesicles to the cell wall, producing the additional layers of CWRAs during extracellular freezing. In pollen tubes of higher plants, Ca^2+^ was reported before as an important interface between the cytoplast and the cell wall, indicating that higher concentrations of Ca^2+^ support the rigidity of the cell wall (Hepler and Winship, 2010), which is in good agreement with our findings. Moreover, the thylakoid structure of the chloroplast was even more dilated during extracellular freezing stress (−2°C and −4°C) compared to chilling stressed cells at 4°C and cisternae of dictyosomes degraded when freezing was induced. It seems that low temperatures do not harm structure and function of dictyosomes of *Klebsormidium* until freezing is induced and cisternae started to degrade.

Cold temperature stress goes along with profound changes of the physiology in *Klebsormidium.* The polarographic oxygen measurements performed in the present study, indicated that the observed ultrastructural alterations of *Klebsormidium* during chilling stress at 4°C were accompanied by a statistically significant (*p* < 0.05) decrease of apparent and gross photosynthesis after 1 h of this stress (Figures 1A,B). In contrast, respiration was significantly (*p* < 0.05) reduced only after 24 h of chilling stress (Figure 1A). Nevertheless, respiratory capacity was still maintained when aggregation of mitochondria occurred after 24 h of chilling stress. Similar ultrastructural and physiological effects were observed in the unicellular freshwater alga *Micrasterias denticulata* during ionic stress, suggesting that fusion and aggregation of mitochondria and the accompanying interconnection of the respiratory chains counteract the impact of the stressor (Steiner et al., 2018). After 3 weeks of chilling stress of *Klebsormidium* at 4°C, respiration and gross photosynthesis were not significantly different from the controls, indicating a possible acclimation to the stress. This goes along with observations on the freezing tolerance of *Klebsormidium flaccidum*, which was substantially increased by cold treatment (Nagao et al., 2008). When *K. flaccidum* cells were transferred from 18°C to −10°C only 15% survived, but with an acclimation at 2°C for 2 days, survival was increased to 55% and after acclimation for 7 days at 2°C to 85%. The physiological explanation for this acclimation can be found in enhanced concentrations of soluble sugars (glucose and sucrose), a putative glycoside and amino acids, including gama-aminobutyric acid (GABA) accumulated in this alga during cold acclimation (Nagao et al., 2008). Similar observations were made by Miguez et al. (2020), indicating an initial increase of sucrose during cold acclimation at 5°C.

The development of the AFU method (Buchner et al., 2020) allowed the preparation of frozen *Klebsormidium* filaments for further TEM investigations. To our knowledge, these are the first ultrastructural images and tomograms of frozen algal samples prepared prior to high pressure freeze fixation (HPF), together with recently published data of algal or plant tissue (Buchner et al., 2020). In our opinion, it is important to analyze 3-D ultrastructure of cells with different 2-D and 3-D electron microscopic methods as performed in previous studies before (Wanner et al., 2013; Steiner et al., 2018). This provides the opportunity to correlate ultrastructural findings with physiological data to obtain insights in the interplay between function and structure of organisms.

In this study, we demonstrated additional cell wall layers that increased in size from chilling to freezing stress. While their formation started as small CWPs during chilling stress, they developed into large CWRAs particularly in the corners of the cell. We therefore suggest that the CWRAs support the stabilization of the xylan containing cell wall of *Klebsormidium* to prevent a collapse of the cell due to extracellular freezing. Furthermore, individual thick cross wall areas contain higher amounts of callose to support the flexibility of that specific part of the cell wall. This corroborates earlier findings by Herburger and Holzinger (2015), who reported an increase of the callose content after desiccation. This could help the alga to counteract the physical pressure of freeze-dehydration. Despite substantial efforts, staining with 15 different monoclonal antibodies (Table 1) to investigate the composition of CWRAs, we could not elucidate their composition. Additionally, our results show that the alga *Klebsormidium* is capable of acclimation to chilling stress after 3 weeks at 4°C by values of respiration and photosynthesis that were statistically not different from the 20°C control cells. This acclimation is accompanied by an aggregation of mitochondria, dilation of thylakoids and a statistically significant (*p* < 0.05) enlargement of ER cisternae. During freezing stress, organelle aggregation increases, ER cisternae enlarge even more and were found in close vicinity to the corner area of the cell wall. The interplay of stabilization and rigidity (CWPs and CWRAs) and flexibility (increase of callose content in individual cross walls), together with organelle aggregation maintains metabolic functions during chilling and freezing stress in *Klebsormidium* down to −4°C. It was the intention of the present study to investigate the structural and physiological changes induced by chilling and freezing stress at temperatures *Klebsormidium* is capable of tolerating. Future studies could elucidate the limits of freezing tolerance in *Klebsormidium*, and in which way cells can benefit from hardening.

## Data Availability Statement

All datasets generated for this study are included in the article/Supplementary Material.

## Author Contributions

PS conducted most of the experiments including experimental freezing, 3-D electron microscopy, and immuno-TEM. SO performed 2-D electron microscopy. GW performed FIB-SEM. OB constructed the freezer. UL-M designed and superviesed the study. AH supervised the study and supplied *Klebsormidium*. PS wrote the draft manuscript. UL-M and AH wrote and edited the manuscript. All authors contributed to the article and approved the submitted version.

## Conflict of Interest

The authors declare that the research was conducted in the absence of any commercial or financial relationships that could be construed as a potential conflict of interest.
