# Supplementary material for: Cell Wall Reinforcements Accompany Chilling and Freezing Stress in the Streptophyte Green Alga Klebsormidium crenulatum
Source: Front Plant Sci. 2020 Jun 24;11:873. doi: 10.3389/fpls.2020.00873 (PMC7344194; doi:10.3389/fpls.2020.00873)
Supplement: Supplementary file 1 [file Data_Sheet_1.PDF]

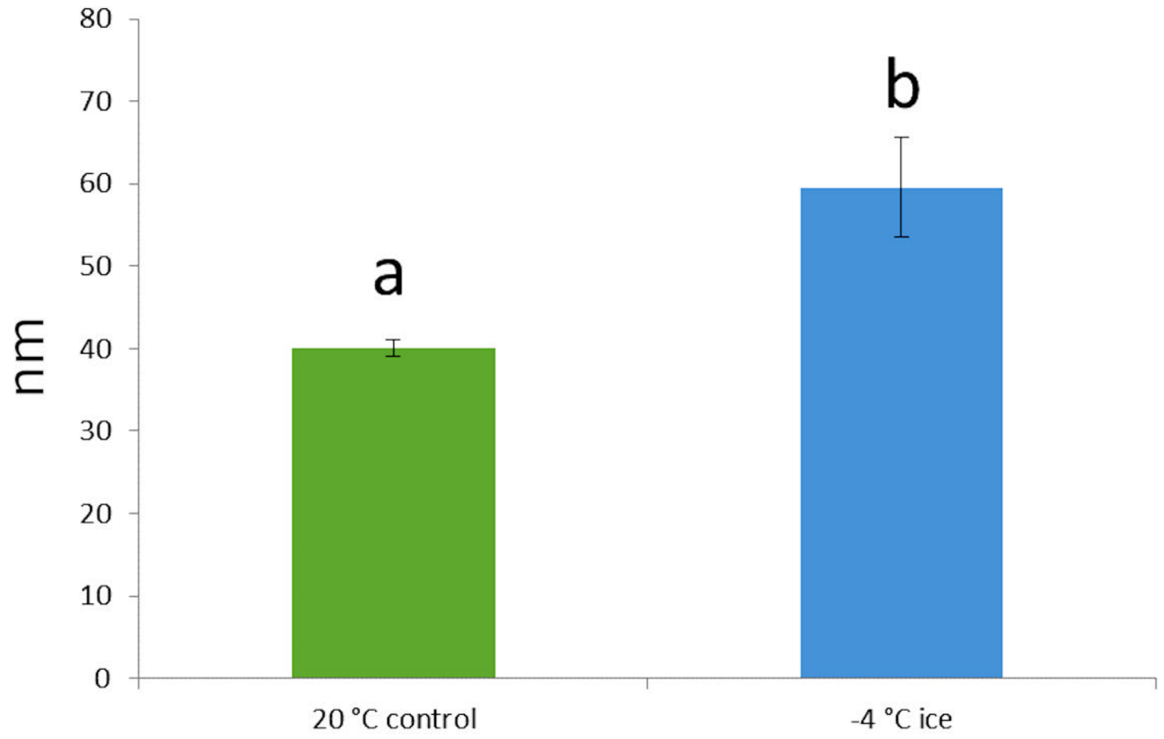

Supplementary Figure 1. Bars represent means of three biological replicates (n=3) with displayed standard error (line). The letters “a” and “b” display significant differences between mean values ($p<0.05$; t-test for two independent samples).
